# A study of visceral leishmaniasis in owned dogs with dermal lesions in Mashhad area, Khorasan Razavi province

**Published:** 2016-03-15

**Authors:** Sadaf Sabzevari, Gholamreza Razmi, Abolghasem Naghibi, Javad Khoshnegah

**Affiliations:** 1 *Department of Pathobiology, Faculty of Veterinary Medicine, Ferdowsi University of Mashhad, Mashhad, Iran; *; 2 *Department of Clinical Sciences, Faculty of Veterinary Medicine, Ferdowsi University of Mashhad, Mashhad, Iran.*

**Keywords:** *Dog*, *Iran*, *Leishmania infantum*, Mashhad

## Abstract

Dog is the main reservoir host of visceral leishmaniasis in Iran. The present study was carried out to investigate visceral leishmaniasis in owned dogs with dermal lesions in Mashhad, Khorasan Razavi province. Thirty- nine owned dogs with dermal lesions were selected. During study, four of dogs were euthanized. The dermal smears, blood and tissue samples were collected and examined using parasitological, serological and molecular methods. A total of 39 examined dogs, leishman bodies were microscopically detected in 33.30% (13/39) of dermal smears. The sera samples were tested by indirect immunofluorescent antibody test (IFAT). Antibody against *Leishmania infantum* was detected in 26.00% (10/39) dogs. According to semi-nested PCR, DNA of* Leishmania infantum* was detected in 2.50% (1/39) of blood samples and in 75.00 % (3/4) of different tissues of euthanized dogs. BLAST analysis of the sequenced samples indicated a 99.00% similarity with kDNA of *Leishmania infantum*. Based on the results, it is concluded that visceral leishmaniasis due to *L. infantum* is distributed among household dogs of this area and it needs more surveillance to control the disease by public health and veterinary authorities.

## Introduction

Zoonotic visceral leishmaniasis (VL) is one of the major health problem and is an important disease in human. The disease is caused by *L.infantum* in the Mediterranean region and Middle East.^1^ Among the members of canidae family, domestic dogs (*Canis familiaris*) are the major reservoir hosts of the Mediterranean type of visceral leishmaniasis.^2^ *Leishmania *spp*.* are transferred by sand flies, mainly by the genera *Phlebotomus *and *Lutzomyia*.^1^ The prevalence rates of canine leishmaniasis has been reported between 10 and 70.00% in Mediterranean area by employment of enzyme-linked immunosorbent assay (ELISA)and analysis of the small subnunit ribosomal RNA genes employing the PCR methods.^3^ The association of human visceral leishmaniasis with presence of infected dogs has been reported in almost any patient in the Mediterranean area.^2^ In 1913, the first case of canine leishmaniasis was reported from Tehran^4^ and subsequently, visceral leishmaniasis were reported from man and dog in Mazandaran province, Iran.^5^ So far, Canine visceral leishmanioasis caused by *L. infantum* have been reported in dogs and other canidae family such as jackals and foxes from the north-west and southern parts of Iran.^6^^-^^9^ The seroprevalence of canine leishmanioasis was determined 7.50% and 8.60% of dogs in Mashhad area.^10^^,^^11^

The present study was carried out to investigate zoo-notic visceral leishmaniasis using direct impression smear, IFAT and semi-nested PCR in a population of owned dogs with cutaneous lesions in Mashhad area, an endemic area of urban cutaneous leishmaniasis in the northeast of Iran.

## Materials and Methods

**Field study area. **The study has been done from February 2011 to February 2012 in Mashhad area, the capital city of the Khorasan Razavi province, northeast of Iran with mountainous climate. This area is classified as semi-arid with cold winters and moderate summer.

**Animals and blood sampling. **The owned dogs belonging to different breeds, ages and genders were examined for clinical signs of the disease in clinic of small animal internal medicine of the Faculty of Veterinary Medicine, Ferdowsi University of Mashhad. Thirty- nine suspected owned dogs with cutaneous lesions were selected for this study. Five mL of blood samples in venouject tubes with and without EDTA were taken from cephalic vein of each dog. The samples were properly labeled and transferred, under cold condition, to parasitology laboratory of Faculty of Veterinary Medicine in the appropriate containers. The plain tubes of blood samples were centrifuged at 6000 rpm for 5 to 10 min at the room temperature, the separated serum in 1.5 mL labeled microcentrifuge and were stored at – 20 ˚C for IFAT and molecular analysis.

**Tissue fragments. **During the study, 4 owned dogs were euthanized based on the method of protocol of American Veterinary Medical Association guideline for the euthanasia of animals.^12^ Briefly, after premedication with intramuscular 0.1 mg kg^-1^ acepromazine (Alfasan, Woerden, The Netherlands), anesthesia was induced with combination of intravenous injection of 6 mg kg^-1 ^Ketamine hydrochloride (Alfasan) and 0.2 mg kg^-1 ^diazepam (Caspian Pharmaceutical Co., Rasht, Iran) and then intracardiac injection of saturated magnesium sulphate. After euthanasia, fragments of popliteal lymph nodes, spleen, liver and skin were collected. Samples were stored at – 20 ˚C for DNA extraction.

**Parasitological study. **Parasitological examinations were performed on 39 symptomatic dogs with dermal lesions. Smears were prepared from any skin lesion. All of the prepared smears were fixed with absolute methanol, stained with Giemsa stain 10% and examined microscopically for the demonstration of amastigote forms of *Leishmania*.

**Serology. **In the present study, the indirect immuno-fluresecent antibody test (IFAT) was used as sero-diagnostic tool. The *L. infantum *antigen (MegaCor, Hörbranz, Austria) has been used for this study. For each reaction, 10 µL of serum dilution (1:50) in PBS were added over the slide holes. The slides were incubated in a humid home at 37 ˚C for 30 min. The slides were washed for 5 min in PBS. The conjugate was diluted to 1:200 in PBS with 0.025% Evan’s Blue and then 15 µL of this solution was placed over the slide holes. The incubation and washing steps were repeated once, as outlined above. Slides were mounted with buffered glycerin, covered with a cover slip and read under a fluorescent microscope (Model BX-FLA; Olympus, Tokyo, Japan) equipped with a 100 W mercury lamp with 400× magnifying power. The serum dilution showing an evident yellow-green fluorescent signal upon microscopic examination was accepted to be positive IFAT titer.

**DNA extraction. **DNA was extracted from blood and tissues samples by DNA-plus extraction Kit (Cinnagen, Tehran, Iran) according to the manufacturer's instructions. The extracted DNA was stored at – 20 ˚C until it could be tested for *leishmania* kDNA.

**Polymerase chain reaction (PCR). **An assay based on the semi nested PCR was used foramplification of variable area of the minicircle kDNA by the method of Aransay *et al*.^13^ Negative and positive controls were performed for each experiment. After amplification, the PCR products were subjected to 2% agarose gel electrophoresis, stained with ethidium bromide and photographed under ultraviolet light.

**Sequencing. **Positive products were selected and sequenced in the facilities of Bioneer Inc. (Seoul, South Korea). Sequences were analyzed using NCBI BLAST, National Institutes of Health, USA.^14^

**Statistical analysis. **The relationship between infection rate and risk factors such as age, gender was analyzed by the chi-square test. The association and agreement between three diagnostic methods were assessed by Cohen’s kappa tests. The agreement between the different tests was expressed as k-value. The agreement as: poor if k-values between 0.2 and 0.4, moderate if k-values between 0.4 and 0.6, substantial if 0.6 and 0.8 and good if it exceeds 0.8.^15^ Statistical analyses were performed with the statistics package SPSS (Version 17.0; IBM, New York, USA).

**Ethical consideration. **All protocols and methodologies were revised and approved by the Ethical Committee at Ferdowsi University of Mashhad, Mashhad, Iran. 

## Results

**Fig. 1:**
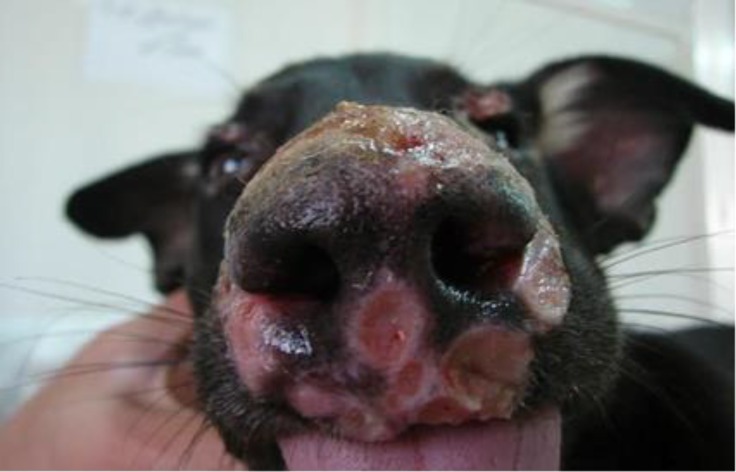
Erosion and ulceration on the lip, nose and around the eye in a German Shepherd dog with leishmaniasis.

**Fig. 2:**
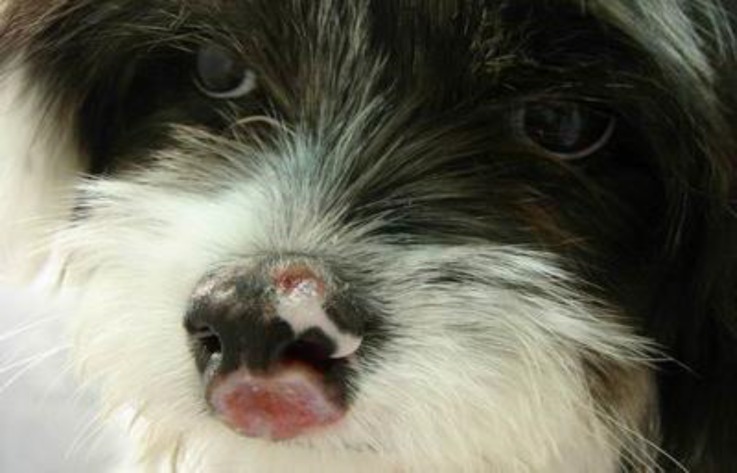
Ulceration on the nose and lip in a Terrier dog with leishmaniasis.

**Fig. 3:**
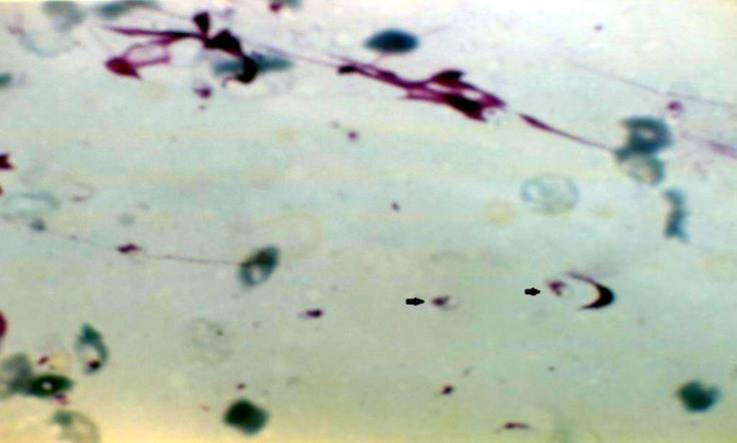
Leishman bodies (black arrows) in dermal lesion of the infected dog (Giemsa stain, 1000×).

**Fig. 4:**
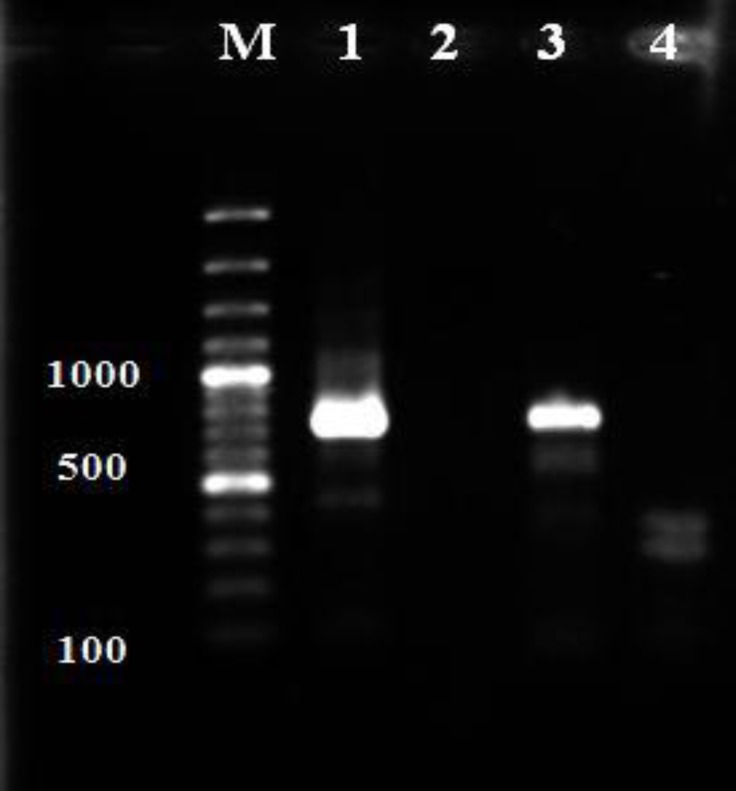
Agarose gel electrophoresis of PCR products from blood samples DNA of dogs. Lane M: standard marker (100 bp). Lane 1: Positive control (720 bp), Lane 2: Negative control, Lane 3: Positive sample, Lane 4: Negative sample

The dogs had cutaneous lesions in different parts of body such as nose, eye, ear, muzzle, mouth, and belly and between the fingers (Figs.1 and 2, Table 1). Leishman bodies were microscopically observed in 33.30% (13/39) of dogs (Fig. 3). Antibodies against *leishmania* *infantum* were also showed in 25.50% (10/39) of dogs (Table. 1). DNA of *leishmania infantum* was detected in one blood sample 2.50% (1/39) of dogs by semi nested- PCR (Fig. 4), while three skin samples 75.00% (3/4), two liver, spleen and lymph nodes 50.00% (2/4) of euthanized dogs were PCR positive (Tables 2 and 3). A poor agreement with a significant difference was observed between microscopic examination with IFAT and PCR methods. The association of risk factors (age, gender and breed) with frequency of *Leishmania* infection in household dogs were analyzed, only the seroprevalence of *leishmania *infection in the age group of 1 to 2 years was significantly higher than other age groups (*p *< 0.05) (Table 4). The sequenced PCR products were found 99.00% identical to *Leishmania infantum* kinetoplast partial minicircle DNA, strain MHOM/ES/97/ LLM-710, clone 591 Sequence ID: emb|AJ275334.1| by BLAST analysis.

**Table 1:** The results of microscopy, IFAT and semi nested-PCR to detect *Leishmania *spp. in 39 owned dogs with cutaneous lesions.

**No.**	**Age (year)**	**Gender**	**Breed**	**Clinical signs**	**Results of laboratory examination**		
					**Microscopy**	**Serology**	**PCR**
**1**	< 1	F	Terrier	Ulcer on the nose and around the eye	–	–	–
**2**	1	M	Mixed	Epistaxis, Ulcer on the nose	+	–	–
**3**	1	F	Terrier	Ulcer on the nose and front paw	+	+	–
**4**	12	F	Mixed	Ulcer on the nose		+	–
**5**	8	M	Mixed	Face and abdomen	+	+	–
**6**	5	F	Mixed	Face and abdomen	+	+	–
**7**	1	F	Terrier	Ulcer on the nose and around the eye	+	+	–
**8**	3	M	German Shepherd	Ulcer on the nose, pinna and around the eye	+	–	–
**9**	3	F	Mixed	Ulcer on the nose, pinna and around the eye	+	–	–
**10**	5	M	Terrier	Ulcer on the nose, pinna and penis	–	–	–
**11**	<1	F	German Shepherd	Ulcer on the nose and around the eye	–	–	–
**12**	<1	F	Spitz	Face and abdomen	–	–	–
**13**	1	M	Pekinese	Face and abdomen	–	+	–
**14**	1.5	F	Shih Tzu	Face and abdomen	+	+	–
**15** *	5	F	Terrier	Ulcer on the nose, pinna and around the eye	–	+	–
**16** *	1.5	M	Doberman	Epistacsis, Ulcer on bridge of the nose	–	–	–
**17**	2	M	German Shepherd	Face and abdomen	–	–	–
**18**	2.5	M	German Shepherd	Face and abdomen	–	–	–
**19**	2.5	M	German Shepherd	Face and abdomen	–	–	–
**20**	1	F	Spitz	Face and abdomen	–	–	–
**21**	1.5	M	Terrier	Face and abdomen	–	+	–
**22**	<1	M	Terrier	Face and abdomen	–	+	–
**23**	2	F	Spitz	Face and abdomen	–	–	–
**24**	3	F	Terrier	Ulcer on the nose	+	–	–
**25**	2	F	German Shepherd	Face and abdomen	–	–	–
**26**	2.5	M	German Shepherd	Face and abdomen	–	–	–
**27** *	2	F	Doberman	Ulcer on the nose and around the mouth	+	–	+
**28**	<1	M	German Shepherd	Face and abdomen	–	–	–
**29**	4	F	German Shepherd	Face and abdomen	–	–	–
**30**	3	M	Terrier	Ulcer on the nose and pinna	+	–	–
**31**	1.5	F	Terrier	Ulcer on the nose, pinna and around the eye	+	–	–
**32**	3	M	Doberman	Face and abdomen	–	–	–
**33**	3	F	Dachshund	Face and abdomen	–	–	–
**34**	3	F	Mixed	Face and abdomen	–	–	–
**35** *	<1	M	German Shepherd	Ulcer on the nose and around the mouth	+	–	–
**36**	2	M	German Shepherd	Face and abdomen	–	–	–
**37**	<1	M	German Shepherd	Face and abdomen	–	–	–
**38**	3	M	Doberman	Face and abdomen	–	–	–
**39**	<1	F	Rottweiler	Face and abdomen	–	–	–
**Total **					13 (33.30%)	10 (25.50%)	1(2.50%)

F: Female, M: Male.

* Euthanized dogs.

**Table 2:** Comparison the results of *Leishmania *spp*.* detection in household dogs with different methods

**Microscopy**	**Total**	**Serology**			**PCR**	
		**Positive No. (%)**	**Negative No. (%)**		**Positive No. (%)**	**Negative No.**
**Positive**	13	5 (38.00)	8 (62.00)		1(7.60)	12(92.00)
**Negative**	26	5(19.00)	21(81.00)		0	26 (100)
**Total**	39	10(25.60)	29(74.00)		1(2.50)	38 (97.00)

**Table 3:** Comparison the results of *Leishmania* spp. detection in euthanized owned dogs with different methods

**Microscopy**	**Total**	**Serology No.**		**PCR No.**							
				**Liver**		**Skin**		**spleen**		**Lymph node**	
		**+**	**-**	**+**	**-**	**+**	**-**	**+**	**-**	**+**	**-**
**Positive**	2	0	2	1	1	2	0	1	1	1	1
**Negative**	2	1	1	1	1	1	1	1	1	1	1
**Total**	4	1	3	2	2	3	1	2	2	2	2

**Table 4:** Frequency of *Leishmania *spp*. *infection in owned dogs with respect to risk factors

**Risk factor**	**Microscopy**		***p*** **-value**	**Serology**		***p*** **-value**	**Total**
	**Positive No (%)**	**Negative**		**Positive No (%)**	**Negative**		
**Age (year)**			*p *> 0.05			*p* < 0.05	
** <1**	1(12.50)	7(87.50)		1(12.50)	7(87.50)		8
** 1-2**	5(55.00)	4(44.00)		6(66.60)	3(33.00)		9
** >2**	7 (31.80)	15(68.00)		3(13.50)	19(86.00)		22
**Sex**			*p* > 0.05			*p* > 0.05	
** Male**	5(26.00)	14(73.60)		4(21.00)	15(78.90)		19
** Female**	8(40.00)	12(60.00)		6(30.00)	14(70.00)		20
**Breed**			*p* > 0.05			*p* > 0.05	
** Terrier**	5 (50.00)	5(50.00)		5(50.0)	5(50.00)		10
** German Shepherd**	2 (16.60)	10(83.00)		0(0)	12(100)		12
** Mixed**	4 (66.60)	2(33.00)		3(50.0)	3(50.00)		6
** Doberman**	1(30.00)	3(70.00)		0(0)	4(100)		4
** Other breed**	1(14.00)	6(85.70)		2(28.6)	5(71.00)		7
**Total**	13(33.30)	26(66.60)		10(25.60)	29(74.40)		39

## Discussion

Visceral leishmaniasis (VL) is one of the most important diseases seen in different geographical locations of Iran and domestic dogs are the principal reservoirs of *leishmania infantum* in the endemic areas of Iran.^16^

Cutaneous lesions are the most common manifestation of canine leishmaniasis in dogs that may be seen along with other clinical signs.^17^ The examined dogs had cutaneous involvement on the face and other areas of body. The diagnosis of canine leishmaniasis based on clinico-pathological abnormalities is difficult and needs specific laboratory tests.^16^ Frequency of *Leishmania* infection were detected in 13 (33.30%) by microscopy and 10 (25.50%) by IFAT. Among of microscopy positive dogs, 8 (62.00%) of dogs were seronegative. The report of the false seropositive and seronegative results is not uncommon for visceral leishmaniasis diagnosis. So far, a few false positive due to cross-reactivity with other parasitic diseases and false negative in early or subclinical cases have been reported.^18^^,^^19^ It seems that the obtained results in this study may be attributed to benign leishmaniasis due to *L. infantum* that is characterized by papular dermatitis with absence or low humoral immunity and a predominant parasite specific cellular immunity.^20^ Some of mentioned dogs had ulcerative dermatitis without any papular dermatitis and may be infected with* L. tropica* the main causative agent of urban cutaneous leishmaniasis in Khorasan province, Iran.^21^ So far *L. tropica* infection has been reported in a few cases of dogs in Iran^22^^,^^23^ and other countries.^24^^,^^25^

Our results also showed that three microscopically negative dogs had specific antibodies against *L. infantum*. A possible reason could be the relatively poor sensitivity of microscopy method to detect amastigotes.^26^

The PCR as a sensitive method for diagnosis visceral leishmaniasis have been used in symptomatic and asymptomatic dogs.^27^ We used a semi nested- PCR to detect to k DNA *Leishmania *spp*.* in blood samples and tissues samples of euthanized dogs.^13^

*Leishmania infantum* k DNA was detected only in 1 (2.50%) of blood samples. Although blood sampling is less invasive and easily performed, the results showed that whole blood sample was not suitable to detect *L.infantum* in infected dogs by PCR. Some studies have been shown that that PCR targeting DNA using peripheral blood reduces the sensitivity of PCR test.^28^^-^^31^ In contrary, the above results, k DNA of *Leishmania infantum* was detected in 75.00% (3/4) of skin samples in euthanized dogs. Two euthanized dogs were seronegative. The results confirmed the high sensitivity of PCR for detection of DNA of *leishmania* in tissue samples of seronegative and seropositive dogs. The results also showed that skin samples were better than other tissue samples for detection of DNA by PCR. A study showed that the highest PCR positivity was observed in skin samples of symptomatic dogs.^32^

We found that there were no significant differences between the seroprevalence of *Leishmania *infection in dogs regarding to their gender and breeds. Similar results were found in other studies in Iran ^8^^,^^22^^,^^33^^-^^37^ and other countries.^38^^,^^39^ Some studies reported that the frequency of leishmaniasis in male dogs is higher than female dogs and German shepherd, Boxer and Doberman breeds are at a higher risk than Yorkshire terrier and Poodle breeds.^40^ Some authors attribute this difference to the fact that large breeds live more outdoors.^41^ Our study showed that the seroprevalence of canine leishmaniasis in the age group 1 to 2 years was significantly higher than other groups. One reason may be due to the higher sensitivity of the younger animal rather than older dogs. The results about age as risk factor are very different, Heidarpour *et al* .was found high frequency of leishmaniasis is young dogs,^11^ Fisa *et al* , in adult dogs,^42^ Mohebali *et al *and Haddadzadeh *et al*., in older dogs^22^^,^^37^ and Miranda *et al*. has been reported bimodal distribution with a first peak formed in adult dogs and a second less evident peak representing in old dogs.^40^

In conclusion, the results of present study showed that many of owned dogs were infected by *L. infantum* and prevention measures, such as examining of suspected dogs by sensitive tests and eliminating or treating infected cases are necessary. The results also showed that skin is the best tissue for microscopy and PCR samples. 
